# Delayed diagnosis of hydrocephalus: Negligence of enlarging head in a child by parents

**DOI:** 10.1002/ccr3.7957

**Published:** 2023-09-25

**Authors:** Ahtesham Khizar, Soha Zahid, Pradhumna Kumar Yadav

**Affiliations:** ^1^ Department of Neurosurgery Pakistan Institute of Medical Sciences Islamabad Pakistan; ^2^ Jinnah Medical and Dental College Karachi Pakistan; ^3^ Department of Neurosurgery Janaki Health Care and Teaching Hospital Janakpur Nepal

**Keywords:** cerebrospinal fluid, hydrocephalus, ventriculostomy

## Abstract

A 1‐year‐old girl presented with an overly enlarged head for 5 months. Negligence of parents regarding treatment for this enlarged head is concerning. Early treatment can avoid a lot of complications. Hydrocephalus secondary to aqueductal stenosis was diagnosed after a thorough history, examination, and investigations. Endoscopic third ventriculostomy was performed.

## Graphical abstract

Championing hydrocephalus awareness: One child at a time.

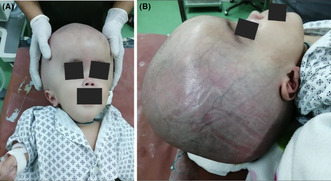

Hydrocephalus is a common but complicated disorder caused by a physical or functional restriction of cerebrospinal fluid flow, which results in ventricular dilation and a larger head. Hydrocephalus affects 1.1 out of every 1000 infants, according to recent estimates.^1^ Infants often have progressive macrocephaly, while children over the age of two usually have signs and symptoms of intracranial hypertension. There are numerous causes of hydrocephalus. Congenital hydrocephalus has been linked to genes that control brain growth and development, most notably involving aqueduct stenosis. Pathological processes that impair ventricular outflow, subarachnoid space function, or cerebral venous compliance can cause hydrocephalus. Shunts and endoscopic approaches are two treatment options that should be tailored to the child.^2^

A 1‐year‐old girl presented to us with an overly enlarged head for 5 months (Figure.1A,B). Negligence of parents regarding treatment for this enlarged head is concerning. Early treatment can avoid a lot of complications in such cases. After detailed history, examination and investigations a diagnosis of hydrocephalus secondary to aqueductal stenosis was made. The child underwent endoscopic third ventriculostomy. (Figure. 2) Such a rare presentation may be underrepresented in well health served communities, and it is encountered in low‐ to middle‐income countries where the access to pediatric neurosurgery is challenging to intercept earlier the enlarging head.

**FIGURE. 1:**
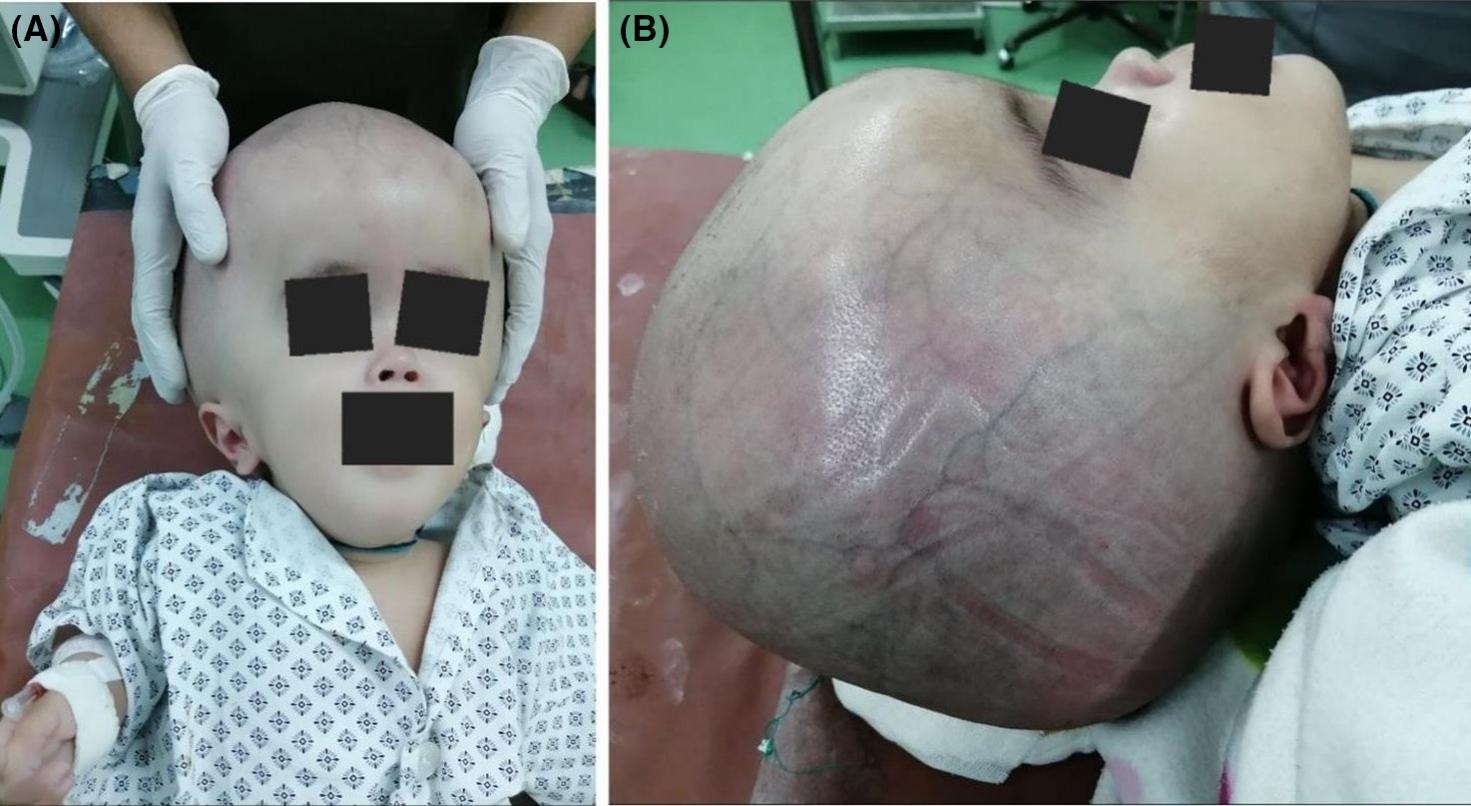
(A and B). A 1‐year‐old girl with macrocephaly. The images illustrate the enlarged head and prominent scalp veins secondary to hydrocephalus.

**FIGURE. 2:**
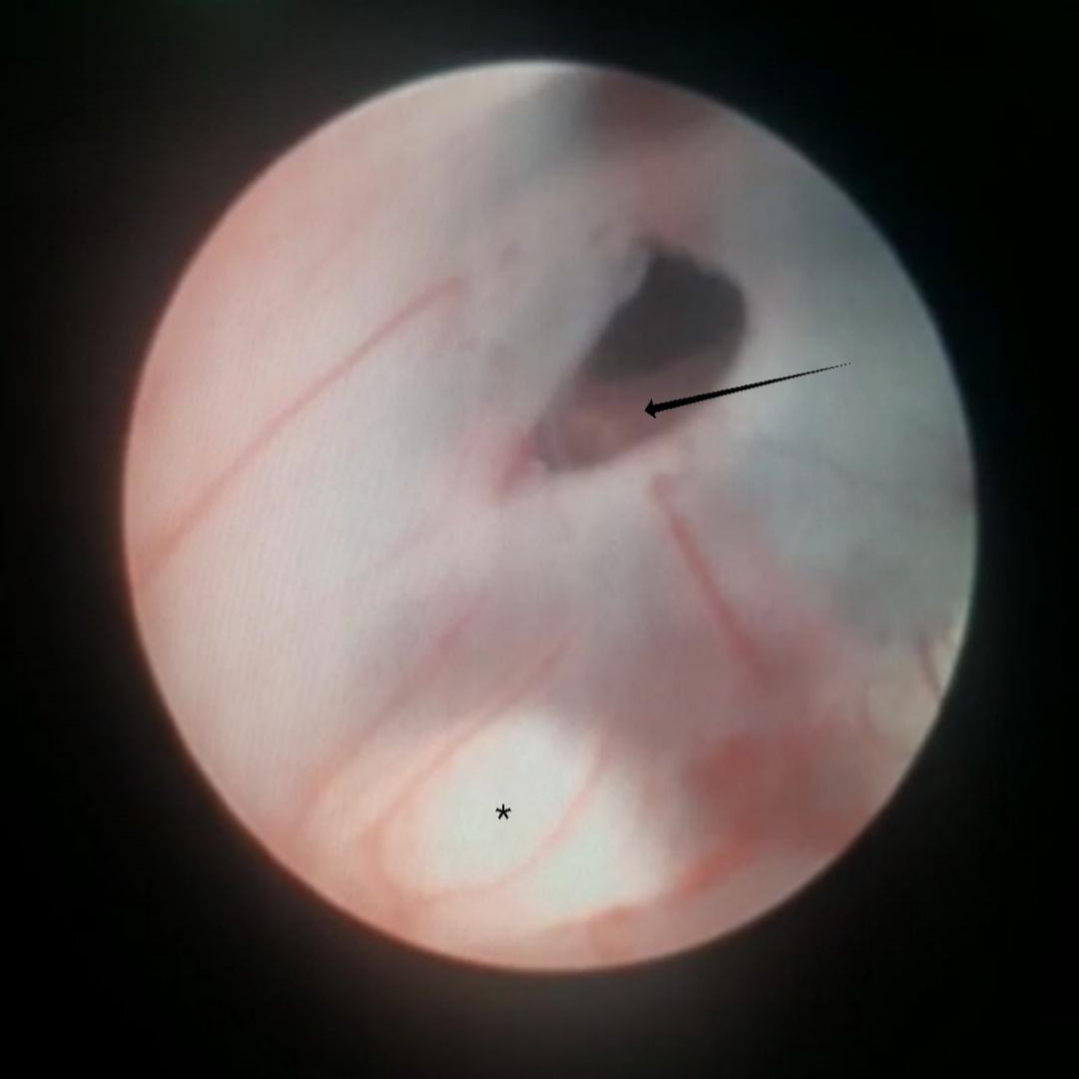
Per‐operative image during endoscopic third ventriculostomy. Arrow represents a basilar artery visible through the stoma. Asterisk indicates mammillary body.

## AUTHOR CONTRIBUTIONS

**Ahtesham Khizar:** Conceptualization; data curation; methodology; writing – original draft; writing – review and editing. **Soha Zahid:** Formal analysis; software; visualization. **Pradhumna Kumar Yadav:** Investigation; methodology; resources; supervision; validation.

## FUNDING INFORMATION

No funding was acquired for this work.

## CONFLICT OF INTEREST STATEMENT

Authors declare no conflicts of interest.

## ETHICAL APPROVAL

Not applicable.

## CONSENT

Written informed consent was obtained from the father for the publication of this case and accompanying images.

## Data Availability

All the data is available within the article.
